# Supplementary material for: MetaRibo-Seq measures translation in microbiomes
Source: Nat Commun. 2020 Jun 29;11:3268. doi: 10.1038/s41467-020-17081-z (PMC7324362; doi:10.1038/s41467-020-17081-z)
Supplement: Supplementary file 10 — Supplementary Data 7 [file 41467_2020_17081_MOESM10_ESM.zip › File2/Confidence_High_Taxonomy/319533_out.krona.html]

Javascript must be enabled to view this page.

members
magnitude
magnitudeUnassigned
count
unassigned
taxon
rank

319533\_out

39

39
superkingdom
2

1239
39
phylum

class
39
186801

186802
39
order

39
family
186803

genus
39
841

166486
species
38

SRS011084\_contig\_number\_34230SRS013158\_contig\_number\_4306SRS013951\_contig\_number\_35811SRS014287\_contig\_number\_687SRS014923\_contig\_number\_12629SRS015065\_contig\_number\_contig-100\_1047.210825SRS015190\_contig\_number\_14320SRS015431\_contig\_number\_15343SRS015578\_contig\_number\_14315SRS015579\_contig\_number\_18119SRS015890\_contig\_number\_14885SRS016335\_contig\_number\_33689SRS017821\_contig\_number\_21737SRS018984\_contig\_number\_30597SRS022137\_contig\_number\_contig-100\_987.132838SRS043001\_contig\_number\_5000SRS043701\_contig\_number\_12189SRS052027\_contig\_number\_16358SRS053335\_contig\_number\_11266SRS054956\_contig\_number\_8520SRS055017\_contig\_number\_26504SRS058070\_contig\_number\_12030SRS063040\_contig\_number\_43961SRS077454\_contig\_number\_12409SRS077641\_contig\_number\_17869SRS098061\_contig\_number\_contig-100\_65.93306SRS104084\_contig\_number\_4785SRS1041130\_contig\_number\_4296SRS105153\_contig\_number\_32447SRS142503\_contig\_number\_16325SRS142781\_contig\_number\_9347SRS143070\_contig\_number\_17421SRS143722\_contig\_number\_15730SRS143780\_contig\_number\_13318SRS147919\_contig\_number\_2246SRS148784\_contig\_number\_27110SRS149181\_contig\_number\_26303SRS149879\_contig\_number\_25654

2049040
species
1

SRS1055069\_contig\_number\_9716
